# Chemosensitivity of MCF-7 cells to eugenol: release of cytochrome-c and lactate dehydrogenase

**DOI:** 10.1038/srep43730

**Published:** 2017-03-08

**Authors:** Rana Al Wafai, Warde El-Rabih, Meghri Katerji, Remi Safi, Marwan El Sabban, Omar El-Rifai, Julnar Usta

**Affiliations:** 1 Department of Biochemistry and Molecular Genetics, Faculty of Medicine, American University of Beirut, Beirut, Lebanon; 2 Department of Anatomy, Cell Biology and Physiological Sciences, Faculty of Medicine, American University of Beirut, Beirut, Lebanon

## Abstract

Phytochemicals have been extensively researched for their potential anticancer effects. In previous study, direct exposure of rat liver mitochondria to eugenol main ingredient of clove, uncoupled mitochondria and increased F_0_F_1_ATPase activity. In the present study, we further investigated the effects of eugenol on MCF-7 cells in culture. Eugenol demonstrated: a dose-dependent decrease in viability (MTT assay), and proliferation (real time cell analysis) of MCF-7 cells, (EC_50_: 0.9 mM); an increase in reactive oxygen species; a decrease in ATP level and mitochondrial membrane potential (MitoPT JC-1 assay); and a release of cytochrome-c and lactate dehydrogenase (Cytotoxicity Detection Kit ^PLUS^) into culture media at eugenol concentration >EC_50_. Pretreatment with the antioxidants Trolox and N-acetyl cysteine partially restored cell viability and decreased ROS, with Trolox being more potent. Expression levels of both anti- and pro-apoptotic markers (Bcl-2 and Bax, respectively) decreased with increasing eugenol concentration, with no variation in their relative ratios. Eugenol-treated MCF-7 cells overexpressing Bcl-2 exhibited results similar to those of MCF-7. Our findings indicate that eugenol toxicity is non-apoptotic Bcl-2 independent, affecting mitochondrial function and plasma membrane integrity with no effect on migration or invasion. We report here the chemo-sensitivity of MCF-7 cells to eugenol, a phytochemical with anticancer potential.

Eugenol, 4-allyl-2-methoxyphenol, is a phytochemical and the main ingredient of clove (*Syzigium aromaticum*) oil^1^. In addition to its uses as a flavoring agent in food products and to add fragrance in cosmetics^2^, it has been widely used in traditional medicine in many Asian countries as an antiseptic, analgesic, or antibacterial^3^ agent in dentistry as cavity filling^4^. Other studies have demonstrated antiviral^5^, antioxidant^6^, antiinflammatory and antiproliferative^7^ effects of eugenol at concentrations lower than 60 *μ*g/ml, although tissue-damaging pro-oxidant effects at a higher concentration (62.1 *μ*g/ml)^8^ have been observed.

The joint FAO/WHO committee on food additives and the Food and Drug Administration^9^ have recognized eugenol as a safe chemical, with no carcinogenic or mutagenic effects^10^. However, despite the reported safety, concentration-dependent adverse side effects of eugenol have been noted^11^,^12^,^13^. Reduced healing process, cytotoxicity and necrosis were observed in eugenol (0.1–1 mM) treated macrophages^14^,^15^, human periodontal ligament cells^16^, osteoblasts^17^, and fibroblasts^18^.

*In vivo* studies of eugenol in a mouse model of skin carcinogenesis have shown down-regulation of c-Myc, H-ras and Bcl-2 expression and the up-regulation of p53, Bax, and active caspase-3 in skin lesions^19^. Other studies have demonstrated a protective effect of eugenol against *N*-methyl-*N*-nitro-*N*-nitrosoguanidine induced gastric carcinogenesis in a rat model^20^,^21^; this protective effect was mediated by the inhibition of NF-κB and modulation of Bcl-2 proteins^20^,^21^.

Studies of the effects of eugenol on various cancer cell lines have reported induction of apoptosis in human melanoma G361 cells mediated by activation of caspases 3 and 6^22^; induction of apoptosis in mast cells through translocation of p53 to the mitochondria^23^; and a decrease in the mitochondrial membrane potential of human leukemia cells (HL60) by activating caspase 3, an increase in reactive oxygen species (ROS) and a reduction in thiol levels^24^. In addition, eugenol modulated growth and cyclooxygenase-2 expression in human colon cancer HT-29 cells^25^, exerted an antiproliferative effect on colon cancer cells (HCT-15 and HT-29)^26^ and inhibited growth of human breast cancer MCF-7 cells in a dose-dependent manner, accompanied by a depletion in GSH and an increase in peroxidation activity^27^.

An *in vitro* study, performed on isolated rat liver mitochondria, documented an uncoupling effect exerted by eugenol accompanied by an increase in F_0_F_1_ ATPase activity^28^.

In the current study, the effect of the aqueous extract of commonly used spices’ on different cancer cells including MCF-7 cells was initially investigated. We then focused on eugenol, the main ingredient of the most potent spice-clove, to explore its underlying mechanism of action. We found that it had a dose- dependent effect on the viability of MCF-7 cells (EC_50_ of 0.9 mM) that is Bcl-2 independent; and that causes release of both cytochrome-c and lactate dehydrogenase (LDH) in the culture media at concentrations greater than 1 mM. A partial protective effect was obtained with Trolox and N-acetyl cysteine but not superoxide dismutase (SOD). Real time cell analysis (RTCA) showed that eugenol reduced the proliferation of MCF-7 cells but had no effect on their invasiveness or migration capability.

## Results

### Effect of different spices on the viability of MCF-7 cells

**Figure 1:**
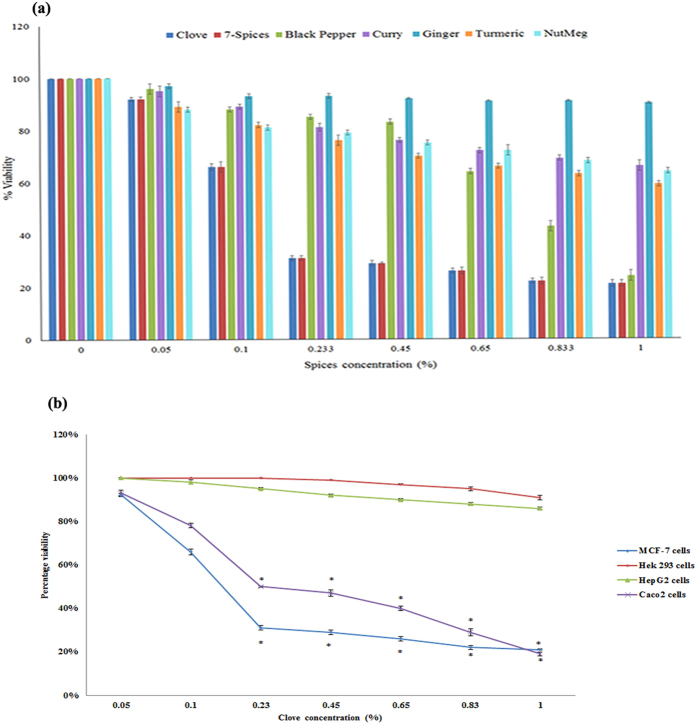
(**a**) The effect of spices on viability of MCF-7 cell line. The seven different spices: “Clove, 7-spices, Black Pepper, Curry, Ginger, Turmeric and Nutmeg” were purchased from Spices and Herbs Store in Lebanon. Each spice was soaked, for 30 minutes, in 50 ml double distilled boiled water, then filtered using a filter paper to prepare a specific % stock solutions that were distributed in small aliquots and stored at −20 °C. All values were tested for normal distribution using Shapiro-Wilkis Test: (Clove: p = 0.30; 7-spices: p = 0.32; Black Pepper: p = 0.192; Curry: p = 0.587; Ginger: p = 0.10; Turmeric: p = 0.735; Nutmeg: p = 0.790). Each value represents mean ± SEM of nine determinations from three different experiments compared to control of vehicle (water) treated cells; *P value < 0.05 was considered significant. (**b**) The effect of clove extract on different cell lines. The effect of aqueous clove extract on cultured HepG2, Hek293, Caco2, and MCF-7 cells were compared. |Cells were treated for 24 hours with varying clove extract concentrations (0.05–1%) following which viability was determined using MTT assay. All values were tested for normal distribution using the Shapiro-Wilkis Test (HepG2: p = 0.33; Hek293: p = 0.172; Caco2: p = 0.117; MCF-7: p = 0.12). Each value represents mean ± SEM of nine determinations from three different experiments compared to control of vehicle (water) treated cells; *P value < 0.05 was considered significant.

Preliminary screening of the viability of MCF-7 cells using the aqueous extract of commonly used spices (0.05–1%) identified clove as most potent spice (Fig. 1a). There was a significant (>50%) decrease in viability following treatment with a 0.23% concentration of clove extract. We also compared the effect of aqueous clove extract on different cell lines. MCF-7 cells were the most sensitive followed by Caco2 cells while Hek293 and HepG2 were insensitive (Fig. 1b). In this study, we investigated the effect of eugenol, the main ingredient in clove, on MCF-7 cells and examined whether it causes the reduction in viability.

### Eugenol decreases the viability of MCF-7 independent of Bcl-2 overexpression

**Figure 2:**
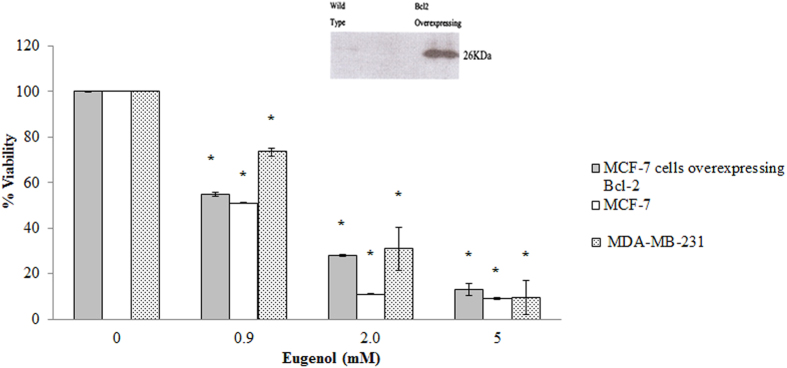
Effect of eugenol on viability of MCF-7 cells, MDA-MB-231 cells and Bcl-2 over expressing MCF-7 cells. All values were tested for normal distribution using the Shapiro-Wilkis Test (p = 0.612). Each value represents mean ± SEM of nine determinations from three different experiments. *P value < 0.05 was considered significant compared to a control.

Treatment with eugenol led to a significant (p < 0.05) and, dose dependent, decrease in the viability of MCF-7 cells with a maximum decrease of 90% at 2.5 mM and an EC_50_ of 0.9 mM (Fig. 2a). The effect of eugenol on the viability of another breast cancer cell line, MDA-MB- 231, was also examined and compared to MCF-7cells. Viability of MDA-MB-231 cells decreased to 75% at 0.9 mM eugenol with an estimated EC50 of 1.6 mM eugenol. At higher eugenol concentrations MDA-MB-231 cells were less sensitive than MCF-7 cells (Fig. 2b).

The possible protective effect of Bcl-2 overexpression in MCF-7 cells was also examined. MCF-7 cells over expressing Bcl-2 that were treated with eugenol exhibited a profile similar to that of MCF-7 cells, with a dose-dependent decrease in the viability of MCF-7 cells over expressing Bcl-2 (Fig. 2b). Overexpression of Bcl-2 in MCF-7 cells was verified by the separation of cell lysates (20 *μ*g) using SDS–PAGE followed by immunoblotting (inset in Fig. 2b).

### Eugenol dissipates the mitochondrial membrane potential in both MCF-7 cells and Bcl-2 overexpressing MCF-7 cells

**Figure 3:**
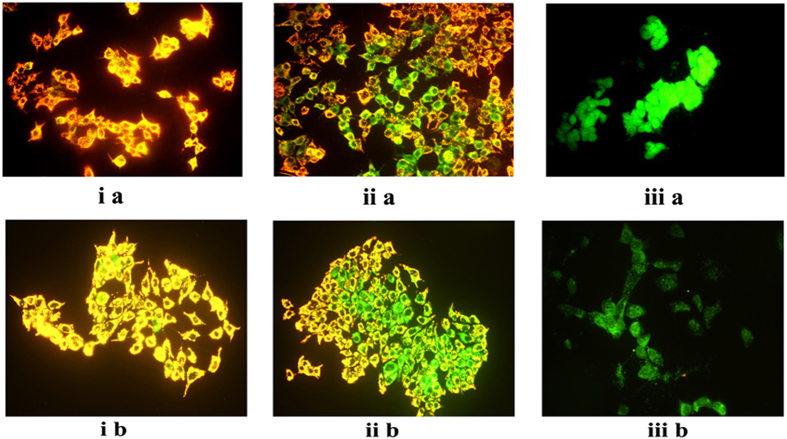
Effect of eugenol on mitochondrial membrane potential of: (**a**) MCF-7 and (**b**) Bcl-2 overexpressing MCF-7 treated cells: (i) control; and eugenol at (ii) EC_50_ (0.9 mM); (iii) 5 mM.

The Mito PT^TM^ JC-1 fluorescent probe was used to monitor the effect of eugenol on the mitochondrial membrane potential of treated MCF-7 cells. Whereas untreated viable cells (control) with coupled mitochondria stained red-orange (Fig. 3a-i), the eugenol (EC_50_)-treated cells stained green and red-orange (Fig. 3a-ii) indicating partial dissipation of the mitochondrial membrane potential; however, when treated with 5 mM eugenol, all cells stained green (Fig. 3a-iii), indicating the collapse of the electrochemical gradient across the mitochondrial membrane.

Bcl-2 overexpressing MCF-7 cells were also sensitive to eugenol treatment (Fig. 3b). Dissipation of the mitochondrial membrane potential was concentration dependent (Fig. 3b-ii and iii) suggesting that Bcl-2 has no protective effect on eugenol-induced cytotoxicity.

Because there was no difference in the response between MCF-7 and Bcl-2 over expressing MCF-7 cells, subsequent experiments were performed only on MCF-7 cells.

### Eugenol decreases intracellular ATP levels in MCF-7 cells and causes LDH release

**Figure 4:**
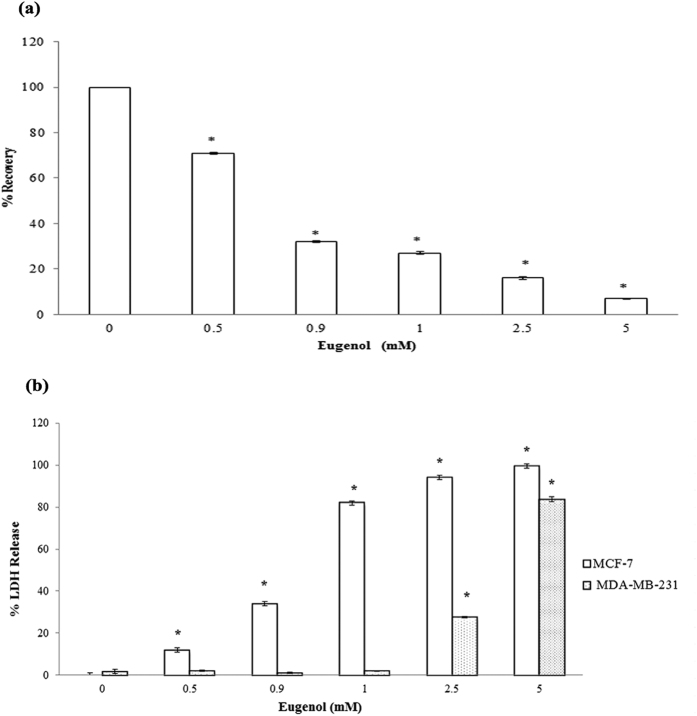
(**a**) Variation in ATP level in eugenol-treated MCF-7 cells. (**b**) Effect of eugenol on LDH release from MCF-7 and MDA-MB-231 treated cells. All values were tested for normal distribution using Shapiro-Wilkis Test: (ATP: p = 0.321; LDH: p = 0.112). Each value represent mean ± SEM of nine determinations from three different experiments; *P value < 0.05 was considered significant compared to control.

Depolarization of the mitochondrial membrane potential may cause a decrease in ATP levels. We therefore, assessed the intracellular ATP levels in eugenol-treated MCF-7 cells. There was a significant (p < 0.05), dose-dependent decrease in ATP levels (Fig. 4a) with increasing eugenol concentrations (0.5–5 mM) resulting in a 68% decrease in ATP level at eugenol’s EC_50_.

We next investigated whether eugenol affects not only the mitochondrial membrane but other membranes as well, such as the plasma membrane. A marker of membrane integrity is the level of LDH released into the media. Eugenol caused a dose-dependent release of LDH of 34% and 99% at its EC_50_ and at 2.5 mM, respectively, indicating disruption of the cell membrane structure (Fig. 4b). By contrast, LDH release was concentration dependent but not time dependent (data not presented). MCF-7 cells treated with eugenol (EC_50_, 0.9 mM) caused a 34% LDH release at 6 hours with no further release occurring after 12 and 24 hr of treatment. On the other hand, there was no LDH release in eugenol treated MDA-MB-231 cells up to 1 mM eugenol treatment; however, at higher concentration, eugenol (2.5 and5 mM) caused significant release in LDH reaching a maximum of 85% at 5 mM, confirming further the higher chemosensitivity of MCF-7 cells to eugenol.

### Eugenol increases ROS levels in MCF-7 cells: a partial protective effect of some antioxidants

**Figure 5:**
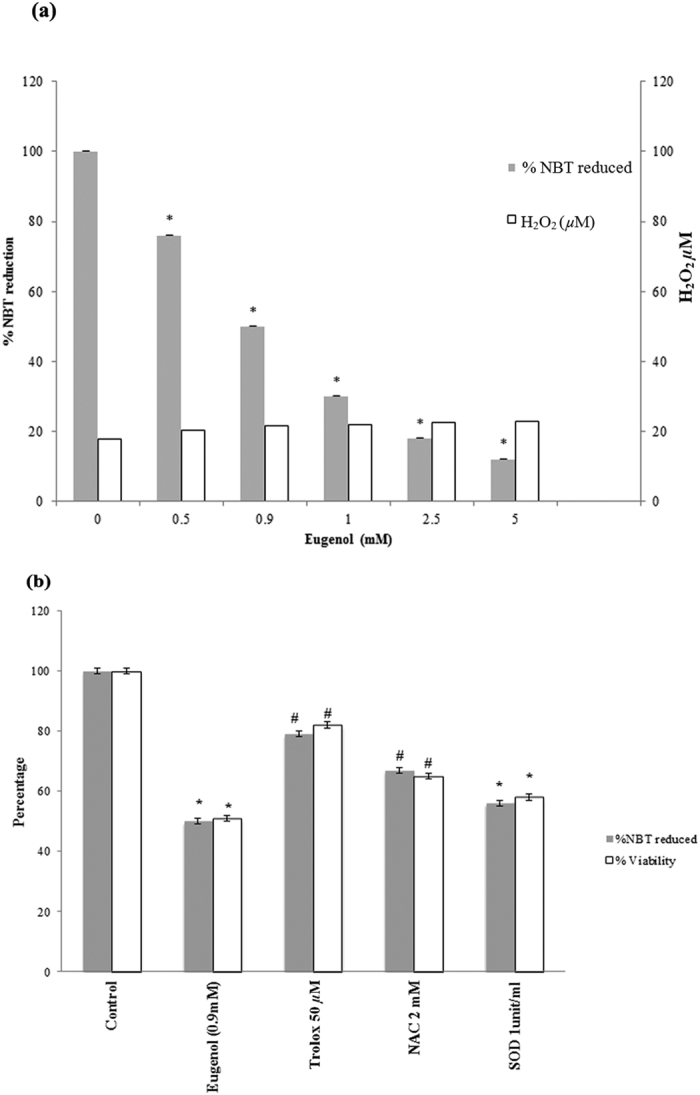
(**a**) Effect of eugenol on NBT reduction and H_2_O_2_ production in MCF-7 cells. All values were tested for normal distribution using the Shapiro-Wilkis Test (NBT reduction: p = 0.553; H_2_O_2_ production: p = 0.205); (**b**) Effect of antioxidants on viability and NBT reduction in eugenol-treated MCF-7 cells. All values were tested for normal distribution using the Shapiro-Wilkis Test (NBT reduction: p = 0.200; viability: p = 0.161). Each value represent mean ± SEM of nine determinations from three different experiments; *P value < 0.05 was considered significant compared to control and eugenol treated cells.

To determine whether eugenol cytotoxicity on MCF-7 cells is mediated by ROS generation, the ability to reduce NBT was compared in control and treated MCF-7 cells. An increase in eugenol concentration resulted in a significant decrease (p < 0.05) in NBT reduction (Fig. 5a) indicating an increase in ROS levels. However, there was no significant variation in H_2_O_2_ levels (Fig. 5a).

The protective effect of anti-oxidants on both viability and ROS levels in eugenol-treated MCF-7 cells was tested. A partial protective effect (p < 0.05) was obtained with Trolox (50 *μ*M) and N-acetyl cysteine (NAC 2 mM), with a restoration in cell viability of up to 82% and 65%, respectively, and an increase in NBT reduction (decreasing ROS) of up to 80% and 66% respectively (Fig. 5b). No significant protective effect was obtained with SOD pretreatment.

### Eugenol effect on apoptotic markers

**Figure 6:**
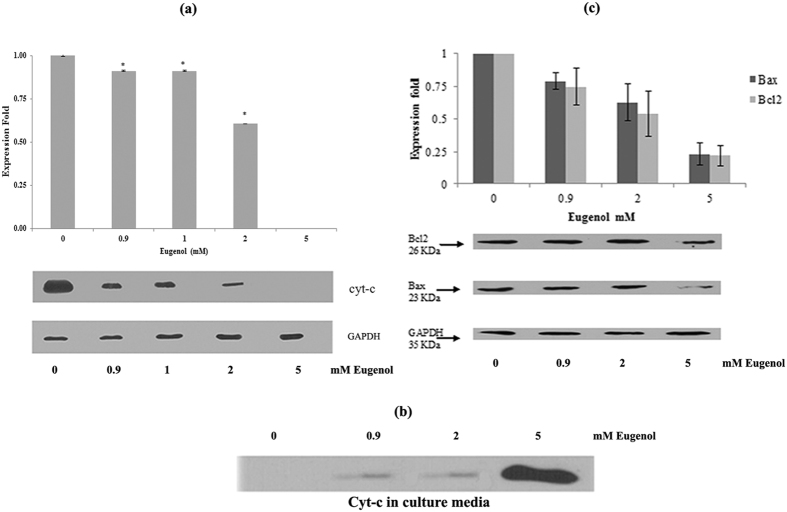
(**a**) Effect of eugenol on expression of cyt-c in MCF-7 treated cells (**b**) Eugenol induces release of cyt-c in culture media; and (**c**) expression of anti- and pro-apoptotic proteins in eugenol-treated MCF-7 cells. Results of densitometry of bands in cropped gels are expressed as mean expression ratio relative to control and are normalized relative to the internal control expression of GAPDH. Each value represent mean ± SEM of nine determinations from three different experiments; *P value < 0.05 was considered significant.

We also investigated the effect of eugenol on expression of apoptotic markers including cyt- c, Bax and Bcl-2. The level of cyt-c decreased significantly (P < 0.05) with increasing eugenol (0.5–2 mM) concentration (Fig. 6a). However, at eugenol concentrations higher than 2 mM, the cyt-c band disappeared completely from the cell lysate and was instead detected in culture media (Fig. 6b). These findings suggest a disruptive effect for eugenol on membrane structures, causing cyt-c release in addition to LDH release.

By contrast, an increasing concentration of eugenol (5 mM) significantly decreased the expression of Bcl-2 and Bax (Fig. 6c) with no significant variation in the relative Bcl-2/-Bax ratio.

### Eugenol decreases proliferation but has no effect on migration or invasion of MCF-7 cells

**Figure 7:**
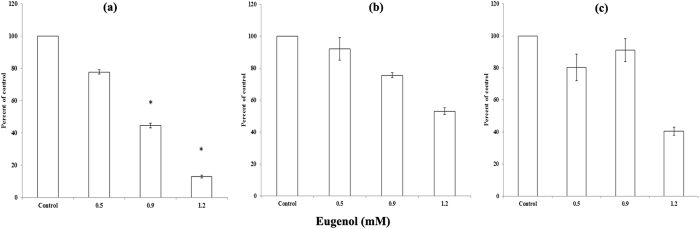
Effect of eugenol on MCF-7 cells using RTCA: (**a**) proliferation (**b**) migration and (**c**) invasion. Each value represent mean ± SEM of three different experiments; *P value < 0.05 was considered significant.

Real time cell analysis (RTCA) was used to measure the proliferation, migration and invasion of treated MCF-7 cells. The interaction of cells with a gold electrode correlated with impedance, which was reported as the cell index. MCF-7 cells were treated for 24 h with eugenol (0.5, 0.9, 1.2 mM) and, then trypsinized, and seeded in RTCA E- and CIM- plates to assess proliferation and migration/invasion respectively. Our results (Fig. 7a) confirmed the inhibition of cellular proliferation by 50% at the EC_50_ of eugenol. There was no change in migration (Fig. 7b) which reveals the motility behavior of eugenol-treated MCF-7 cells. In addition, a nonsignificant change in invasion capability was shown which reflects the ability of MCF-7 cells to interact and degrade the basement membrane (Fig. 7c).

### Eugenol has no apoptotic effect on MCF7 cells: Annexin V/PI and flow cytometry

**Figure 8:**
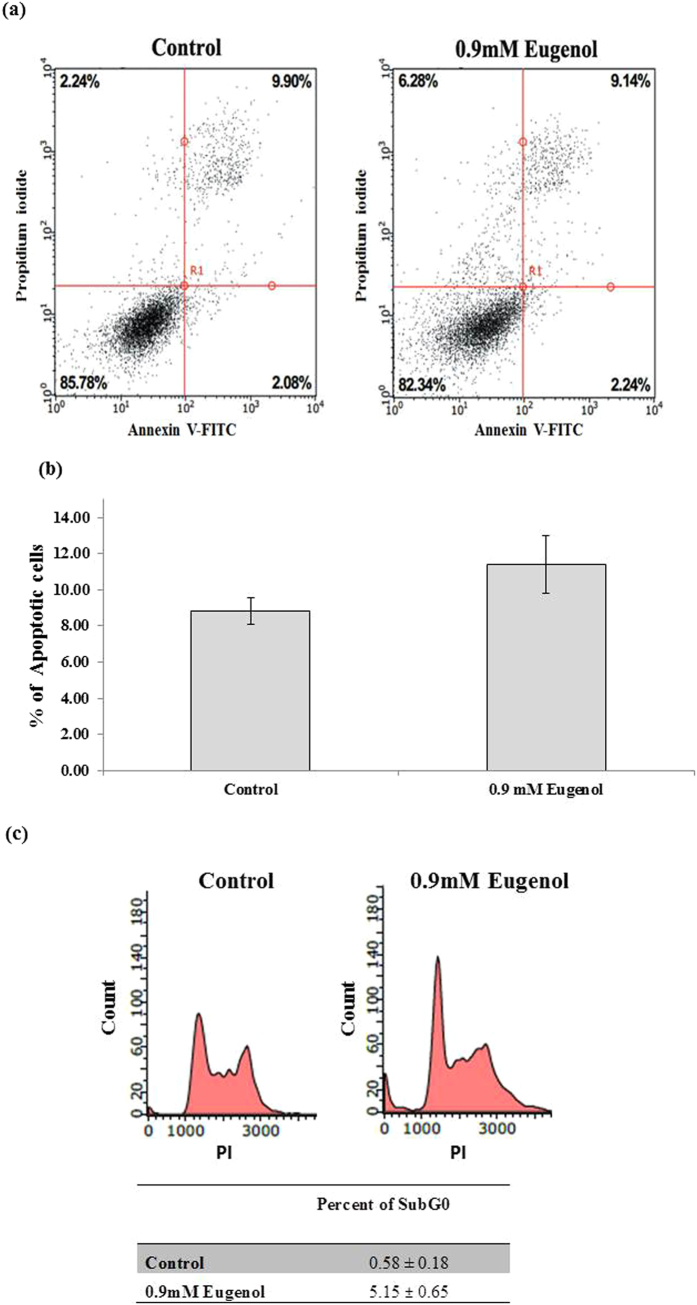
(**a**) Representative dot plots of cell apoptosis for control (vehicle treated: ethanol) and eugenol (0.9 mM) treated MCF-7 cell after Annexin V/PI dual staining. The proportion of dead cells (Annexin V−/PI+), live cells (Annexin V−/PI−), early apoptotic cells (Annexin V+/PI−) and late apoptotic/necrotic cells (Annexin V+/PI+) were measured and compared. (**b**) Percentage of early and late apoptotic MCF-7 cells. Each value represents the mean ± SEM of two different experiments; *P value < 0.05 was considered significant compared to a control cells. (**c**) Representative histogram on the effect of eugenol on MCF-7 cell cycle distribution as measured by PI flow cytometry, compared to control. Each value represents the mean ± SEM of two different experiments; *P value < 0.05 was considered significant.

To determine if eugenol decreases viability by triggering apoptosis we examined the increase in plasma membrane phosphatidylserine exposure using Annexin V. Our findings show no effect on MCF-7 cells treated with eugenol at 0.5, 0.9 and 1.2 mM. A representative image is shown for 0.9 mM eugenol concentration (Fig. 8) with no significant change from the control. Similarly no significant change was observed at higher (1.2 mM) or lower (0.5 mM) eugenol concentrations indicating no apoptosis (data not shown).

Further experiments were performed to validate the Annexin V results, quantifying the sub G0 population in eugenol treated MCF-7 cells using cell cycle analysis flow cytometry. A representative image of cell cycle distribution shows (Fig. 8c) the percent subG0 of: 0.58 ± 0.18%; and 5.15 ± 0.65% for control (vehicle treated: ethanol) and eugenol treated (0.9 mM) respectively. The increase in sub G0 population may be statistically significant but do not represent a biologically significant apoptotic cell death. Our LDH release findings is suggestive of necrotic like mechanism detecting 34% of cytoplasmic LDH after 24 hrs in addition to mitochondrial cyt-c in the culture media.

## Discussion

Natural compounds such as essential oils and spice extracts have been widely used in folk medicine and have been proposed as potential chemo-preventive candidates for cancer treatment^29^. A comparative screening of commonly used spices (clove, 7-spices, black pepper, curry, ginger, turmeric and nutmeg) identified clove as having the greatest potency in decreasing the viability of MCF-7 cells. Likewise, Caco2 cells were sensitive to clove extract while HepG2 and Hek293 cells were not. This is in line with findings from a previous study using human cancer cells^30^ that reported that MCF-7 cells were the most sensitive to clove extract.

Moreover, eugenol, the main component of clove extract^1^, has also been reported to inhibit proliferation of HT-29 cells^26^ as well as the growth of HepG2^31^, HL-60^24^ and MCF-7 cells^27^. The chemosensitivity of cancer cells to eugenol suggests that the compound may have anti-cancer potential.

In this study eugenol decreased the viability of two breast cancer cells: estrogen positive MCF-7, and estrogen negative MDA-MB-231 cells in a concentration dependent manner with MCF-7 being more sensitive. Similar profile was reported on the 2 breast cell lines but at much lower eugenol concentration (1.5–1.7 μM)^32^. The discrepancy in cytotoxicity level is unexplainable, but our findings are in line with the FDA recommended safety level and the commonly used level in dentistry^4^.

Being more sensitive than other tested cell lines, we have explored the targets and modes of action of eugenol on MCF-7 cells. We found that depolarization of the mitochondrial membrane of MCF-7 cells treated with eugenol (0.9 mM, EC_50_) was not compensated for by Bcl-2 overexpression. We also showed that eugenol at concentrations less than its EC_50_ caused significant, yet partially reversible, variations in viability, ROS levels and mitochondrial membrane potential. However, at higher concentration however (1 mM–5 mM), eugenol disturbed the integrity of both the mitochondria and plasma membranes causing the release of cyt-c and LDH into culture media. Similarly eugenol treated MDA-MB-231 cells, resulted in LDH release into the media at higher concentration compared to MCF-7cells, confirming further more sensitivity of the latter.

Humans’ may be exposed to eugenol in cosmetics or as a food additive/or spice, antiseptic^33^ or a local anesthetic commonly used in dentistry^2^,^4^. Although the Food and Agriculture Organization and the World Health Organizations have recognized eugenol as a non-mutagenic safe product, several studies have alluded to its cytotoxicity above a specific concentration and have described it as a possible candidate for cancer treatment. A chemopreventive effect of eugenol against DMBA-induced genotoxicity in MCF-7 cells^34^ and its anticancer potential in various animal models of carcinogenesis and in cancer cell lines have been reported^35^,^36^. Furthermore, exposure of oral soft tissues to eugenol-induced hypersensitivity and local irritation ranging in severity from low grade to serious anaphylactic reactions in rare cases has been documented^11^. Sedation of the dental pulpit^37^, and inhibition of respiration as well as of colony formation in V79 cells^38^ have been reported at eugenol concentrations of 0.1 mM–1 mM.

Other *in vitro* studies using isolated rat liver mitochondria proposed that mitochondria is a possible target for eugenol^28^.

Concomitant with eugenol-induced cytotoxicity, we observed dissipation in the mitochondrial membrane potential (Fig. 3a) leading to a dose-dependent decrease in ATP levels (Fig. 4a). These findings are in line with our previous results showing direct effects of eugenol on isolated mitochondria: a concentration-dependent inhibition of complex-I (NADH-oxidase) of the electron transport chain (ETC); dissipation of the mitochondrial membrane potential (Δψm, DSMP assay); and stimulation of FoF1 ATPase activity^28^.

We also investigated the possible involvement of the intracellular redox state^39^ and/or changes in the expression of pro- or/anti- apoptotic proteins^40^. The cytotoxic effect of eugenol may result from a peroxidation reaction activating eugenol into a reactive electrophile, quinone methide which binds glutathione and intracellular proteins^27^.

Biological ROS include radicals and non-radical species that are generated primarily by the electron transport chain of the mitochondria, flavin oxidases, and cytochrome P450^39^.

Eugenol possesses a pro-oxidant activity^41^, increasing superoxide levels in neutrophils treated with 2 mM eugenol^42^ and causing increase in ROS levels leading to cell death in human promyelocytic leukemia (HL-60) and colon cancer (HCT-15) cell lines^24^,^26^.

In our study, eugenol-treated MCF-7 cells showed a significant dose-dependent increase in ROS levels but not in H2O2 levels discordant with previous studies reporting catalase inhibition by eugenol^43^,^44^. We examined the protective effect of Trolox^45^, the glutathione precursor NAC^46^, and the extracellular superoxide free radical scavenger SOD and found partial restoration of viability and, reduction in ROS levels by both Trolox and NAC but not SOD. A similar protective effect with NAC, but not with catalase or SOD, was reported on eugenol-induced cytotoxicity in human osteoblasts *in vitro*^17^.

The metabolism of eugenol into eugenol quinone methide (EQM) involves both cytochrome -P450 and peroxidases^47^. EQM may bind or conjugate covalently to sulfhydryl groups on proteins or reduced glutathione (GSH), respectively, depleting reductive intracellular levels^48^,^49^,^50^. Depletion of GSH favors the ROS-induced oxidative damage reported in eugenol treated HepG2 cells^31^, MCF-7 cells^27^, human oral mucosal fibroblasts^51^ and cultured rat liver cells^50^. NAC, a precursor of GSH, plays a role in protecting cells against oxidative damage^52^. The protective effect of the antioxidant NAC shown here may be due to its ability to directly scavenge ROS in eugenol-treated MCF-7 cells or indirectly to the promotion of intracellular GSH synthesis^53^.

ROS serves as a subcellular messenger controlling regulatory genes and signal transduction pathways^54^. Dissipation of the mitochondrial membrane potential (Δψm) by eugenol can also decrease ATP levels and increase ROS; both can initiate or transduce apoptotic events leading to variations in anti- and pro-apoptotic proteins expression^55^. Both *in vitro* and *in vivo* studies have demonstrated that eugenol-treated cancer cells have variations in their mitochondrial membranes demonstrated by a decrease in anti-apoptotic Bcl-2 expression and translocation of pro-apoptotic Bax that led to cyt-c release into the cytosol and subsequent activation of caspases-9 and -3^19^,^21^,^24^. In our study, western blot analysis for Bcl-2 and Bax showed a significant variation in protein expression levels at a high eugenol concentration (5 mM). MCF-7 cells over expressing Bcl-2 displayed no protective effect against eugenol cytotoxicity; eugenol treatment of Bcl-2 overexpressing MCF-7 cells resulted in dissipation of the mitochondria membrane potential, indicating that eugenol-induced cell death is Bcl-2 independent. In a previous study, while correlated with loss of mitochondrial membrane potential, no alteration was obtained in apoptotic response of PC-3 cells overexpressing or lacking Bcl-2 to combined treatment of eugenol and 2-methoxy estradiol^1^. Further evaluation of eugenol effects using Annexin V/PI staining revealed no variation in eugenol treated compared to control or vehicle treated MCF-7 cells. While estimation of the subG0 population showed a statistically significant increase (0.58–5.15%) yet its biological contribution to cell death remains insignificant reflecting a DNA fragmentation that may or may not be –apoptotic. Moreover, the significant release of both LDH, and cyt-c in the culture media provides an evidence in support of necrotic –like mechanism rather than apoptotic (Annexin V/PI assay findings).

Release of cyt- c by eugenol into the culture media was dose-dependent. A massive release of cyt-c by eugenol into the culture media was observed at 5 mM of eugenol, indicating rupture of mitochondrial and plasma membranes of the treated cells. The obtained nonsignificant change in invasiveness and migration (RTCA) is concordant with previous report that MCF-7 cells are non-invasive^56^. The transient invasive potential of a sub-population of cells treated with 0.9 mM eugenol ultimately undergo significant cyt-c and LDH release leading to cell death at 72 hours (data not presented). This was further confirmed when higher concentration of eugenol was used (1.2 mM, Fig. 7c).

The ability of the hydrophobic phenolic compound eugenol to penetrate and disrupt the plasma membrane leads to cellular injury^42^, a biomarker of which, is leakage of cytosolic LDH. In our study, significant LDH release occurred in eugenol-treated MCF-7 cells causing damage to the plasma membrane^37^ similar to that in eugenol treated mucosal fibroblasts^51^. Alterations in membrane integrity after eugenol treatment have previously been described in both gram- positive and gram- negative bacteria^57^,^58^ involving the binding of eugenol to bacterial membrane components, pore formation, increases in permeability, and intracellular protein release^57^,^58^. A significant decrease in the viability of *H. pylori* by eugenol identified eugenol as having a potential role in *H. pylori* management^59^.

In conclusion, eugenol treatment at its EC50 caused a decrease in the viability of MCF-7 cells accompanied by an increase in ROS levels, both of which were partially reversed by treatment with NAC or Trolox but not SOD. Moreover, treatment with eugenol caused a decrease in intracellular ATP levels indicating impairment in the mitochondrial function as confirmed by dissipation in the membrane potential and cyt-c release in culture media; disruption of plasma membrane integrity was confirmed by LDH release with no change in cell motility or invasiveness.

Although in our daily life, the clinical application of eugenol is still limited to dentistry (Zinc oxide –eugenol fillings), it is important to note that the chemosensitivity of MCF-7 cells to eugenol occurs at lower concentrations than those previously reported (1–4 mM) in non-cancer cells^60^,^61^ and those commonly used in dental practice^15^,^16^,^17^,^41^. Elucidation of the key players underlying the sensitivity to eugenol and mechanistic differences between normal and cancer cells to eugenol requires further *in vivo* and *in vitro* investigations.

## Materials and Methods

### Materials

The human breast cancer cell line (MCF-7, cat# HTB-22), human liver cancer cell line (HepG2 cat# HB-8065), human colorectal adenocarcinoma cell line Caco2 (cat# HTB-37), and human embryonic kidney cells (Hek293, Cat# CRC-1573), were obtained from American Type Culture Collection (ATCC), Manassas, VA, USA. MDA-MB-231 cells were the generous gift of Marwan El-Sabban originally purchased from ATCC. MCF-7 cells over expressing Bcl-2 were the generous gift of Dr. Ghassan Dbaibo (Department of Pediatrics, Department of Biochemistry and Molecular Genetics, American University of Beirut). Roswell Park Memorial Institute media (RPMI), heat inactivated fetal bovine serum (FBS), Phosphate Buffered Saline (PBS) solution (10X), Penicillin-streptomycin (PEN-Strep mixture), and Trypsin-EDTA 10X-solution were purchased from Lonza.

The ATP Bioluminescence Assay Kit HS II (cat. #: 11 699 709 001), Cell Proliferation Kit I for 3-(4–5 dimethyl thiazol-2yl)-2,5 diphenyl tetrazolium bromide (MTT) assay (cat. #: 11 465 007 001), and Cytotoxicity Detection Kit^PLUS^ (LDH) (cat. #: 04 744 934 001) were purchased from: Roche (Mannheim- Germany). The Hydrogen Peroxide Assay Kit (cat. #. K265-200) was purchased from BioVision (California, USA). The Mito PT^TM^ JC-1 kit (cat# 924) was purchased from Immunochemistry Technologies LLC (MN, USA). The Bradford reagent, nitrocellulose membranes (2 μm) and Enhanced Chemi-Luminescence’s reagent (ECL) Kit (cat# 117-5060) were purchased from Biorad (France).

The antioxidants N-acetylcysteine (NAC) and 6-hydroxy-2,5,7,8 -tetramethylchroman-2-carboxylic acid (Trolox) (Sigma-Aldrich United Kingdom) were freshly prepared as water and ethanol solutions, respectively. Eugenol (4-allyl-2-methoxyphenol) was purchased from Merck (Germany), and p-Nitro Blue Tetrazolium Chloride (NBT) (MP chemicals, CA-USA) was freshly prepared as 1 mg/ml solution in water.

The primary antibodies: Bax rabbit polyclonal IgG (N-20, Sc-493), Bcl-2 mouse monoclonal IgG (Sc-130308), and cytochrome-c (cyt-c) goat polyclonal IgG (C-20, Sc-8385), were purchased from Abcam (MA, USA). The secondary antibodies donkey anti-goat IgG-HRP (Sc-2033), goat anti-mouse IgG-HRP (Sc-2031), goat anti-rabbit IgG-HRP (Sc-2030) were purchased from Santa Cruz Biotechnology (Santa Cruz, CA-USA).

### Cell Treatment

Cells were seeded at a density of 1 × 10^4 ^cells/well/100 μl media in 96-well plates for viability, LDH release, reactive oxygen species (ROS) generation, and hydrogen peroxide production assays. Cells were then incubated for 24 hours in a humidified (5%) CO_2_ incubator at 37 °C and treated for 24 hours with eugenol at final concentrations of 0.1–5 mM.

For intracellular ATP level determination or western blotting, cells were seeded in petri-dishes at a density of 1 × l0^6 ^cells/10 ml media, incubated for 24 hours in a humidified (5%) CO_2_ incubator at 37 °C and then treated for 24 hours with eugenol at different concentrations.

The number of passages of MCF-7 and MDA-MB-231 ranged from 25 to −30, while those of Bcl-2 overexpressing MCF-7 cells ranged from 10 to −12.

### Cell Viability and Cytotoxicity Assays

#### MTT Assay

The effect of eugenol on the viability of MCF-7 cells, MDA-MB-231 cells, and Bcl-2 over expressing MCF-7 cells was determined using the MTT assay kit according to the manufacturer’s instructions. The absorbance of the developed color (595 nm) was measured using an ELISA microplate reader comparing eugenol-treated cells to control cells treated with ethanol (<0.5%).

EC_50_ is the concentration at half maximal response (the point at which inflection in the curve occurs). The absorbance of the control was considered 100% viability. The percentage viability was computed by multiplying the ratio of absorbance obtained of treated cells to that of control by 100.

#### LDH Release

LDH released in eugenol-treated MCF-7 cells and MDA-MB-231 cells was determined using the Cytotoxicity Detection Kit^PLUS^ according to the manufacturer’s instruction. Released LDH in the culture media was coupled to an enzymatic assay yielding a red color, the intensity of which was measured at 490 nm by an ELISA microplate reader. The percentage cytotoxicity expressed as percent release of LDH was determined relative to controls as described by the manufacturer. The kinetics of LDH release was analyzed at EC50 concentration of eugenol (0.9 mM) at 6 and −24 hrs.

### Intracellular ATP levels in eugenol-treated MCF-7 Cells

Intracellular ATP levels in both control and eugenol (0.5 mM to 5 mM) treated MCF-7 cells were measured using the ATP Bioluminescence Assay Kit according to the manufacturer’s instructions. The ATP-dependent light emitted by the luciferase catalyzed oxidation of luciferin was determined on lysates of eugenol-treated MCF-7 cells mixed with 50 *μ*l of luciferase reagent in a white MTP plate. The ATP bioluminescence of the treated cells was measured using a Fluoroskan Ascent FL and compared to control cells treated with ethanol not exceeding 1%.

### Mitochondrial membrane potential of eugenol-treated cells

The Mito PT^TM^ JC-1 kit was used to examine the effect of eugenol on the mitochondrial membrane potential of MCF-7 cells and MCF-7 cells over expressing Bcl-2. Cells were seeded on a cover slip at a density of l × l0^5^ in a 12-well plate and, treated for 24 hours with eugenol (0.9 mM, or 5 mM). Following media aspiration, the cells were incubated for 15 min, at room temperature with 300 *μ*l of 1x Mito PT^TM^ JC-1 stain solution and washed twice. Next the cover slips were mounted on slides and examined (60x magnification) using a fluorescent microscope (Olympus BH2-RFCA) containing long-band-path emission filters (Ex 490 nm and Em >510 nm). Images were captured with an Olympus DP71 camera using the DP Controller acquisition software (Olympus; 2001–2006; 3.1.1.267).

### ROS levels in eugenol-treated MCF -7 cells

The reduction of nitro-blue tetrazolium salt (NBT) into a turquoise colored product was used to indirectly estimate the intracellular ROS levels generated in treated MCF-7 and control cells. NBT (100 *μ*l of 1 mg/ml) was added to treated cells, and then incubated for 1 hour in a CO_2_ chamber at 37 °C. The formed crystals were solubilized by the consecutive addition of KOH (120 *μ*l) and DMSO (140 *μ*l). The intensity of the developed color was measured using an ELISA reader at 645 nm. The percentage of reduction of NBT, which is inversely proportional to the ROS generated, was calculated relative to a control treated with ethanol.

### Hydrogen peroxide levels in eugenol-treated cells

The hydrogen peroxide (H_2_O_2_) released in the culture media of eugenol-treated MCF-7 cells was determined using the Hydrogen Peroxide Assay Kit in the culture media of treated and control cells. Media were collected and different volumes were assayed in a 96 -well-plate according to manufacturer’s instructions. The absorbance was read using ELISA reader at 570 nm, and the H_2_O_2_ level was determined relative to a standard curve.

### Effect of antioxidants and superoxide dismutase (SOD) on viability and ROS in eugenol-treated cells

The protective effect of common antioxidants against eugenol cytotoxicity was investigated. Prior to eugenol (0.9 mM) treatment, MCF-7 cells were pretreated for 2 hours with Trolox (50 *μ*M), NAC (2 mM) or superoxide dismutase (SOD) (1 unit/ml). The cellular viability and generated ROS were determined using MTT and NBT assays, respectively, compared to controls treated only with antioxidants.

### Western blot analysis of Bax, Bcl-2, and Cyt-c

The effect of eugenol on the expression of Bax, Bcl-2 and cyt-c was determined. Control and eugenol-treated cells were washed 1X-PBS and lysed via homogenization in 250 mM sucrose- 10 mM Hepes- 50 mM Tris base buffer pH 7.4 containing triton at 0.001% final concentration. Protein levels were determined using the Bio-Rad protein assay. Following standard protocols, lysates containing 50 *μ*g of protein were loaded, resolved on 12% SDS-PAGE, electro-transferred to a nitrocellulose membrane, immuno-blotted at 4 °C overnight with proper primary antibody (Bax, Bcl-2, cyt-c or GAPDH (1:1000 dilution), washed with TBS-Tween and blotted for 1 h with the appropriate secondary anti-body. The protein bands were visualized using the ECL Kit. The films were exposed using an RP X-OMAT processor (Model M6B) and scanned using an Epson Expression 1680 Pro. The density of bands was quantified using the “ImageJ” program.

To determine whether cyt-c was released from eugenol-treated cells, the culture media of control and treated cells were collected, and lyophilized, and the resultant solid was re-suspended in lysis buffer, separated on 12% SDS-PAGE, and immune- stained as described before.

### Migration, invasion and proliferation of eugenol-treated MCF-7 cells

Quantitative analysis of the effect of eugenol treatment on the migration/invasion of MCF-7 cells effect was performed as described^62^, with minor modifications using a Real Time Cell Analyzer system, RTCA × CELLigence RTCA[A2]DP instrument (Roche, Germany). MCF-7 cells were seeded in 6-well plates (9 × 10^4 ^cells/well) prior to 24 h treatment with 0.9 mM of eugenol, or 1% ethanol (vehicle). Cells were then harvested, counted, re-suspended in 120 *μ*l of serum-free medium and seeded for 24 hours (20 × 10^3 ^cells/well) in the RTCA CIM-plates coated with matrigel for invasion assay and without matrigel for the migration assay. Invasion was monitored by recording cell impedance every 15 minutes for a minimum of 18 hours.

To monitor proliferation cells were seeded at a density of 7000 cells/well, and cultured for 24 hours in the RTCA-E plate, after which they were treated with eugenol (0.9 mM) for 24 hours. Proliferation was monitored by recording cell impedance every 60 minutes for 24 hours.

Using the RTCA × CELLigence system (Roche Applied Science, Indianapolis, IN, USA), the cell impedance created as the cells attached and detached from the gold electrodes in the CIM and E-plates were recorded. This generated a survival curve, computed by the software of a computer system connected to the RTCA that estimated the cell survival or cell index (CI). This CI, correlates directly with the number of cells, was expressed in histograms as % of control, (taking the survival of the control cells as 100%).

### Cell cycle analysis

The effect of eugenol on cell cycle phases of eugenol treated MCF-7 cells was analyzed using flow cytometry. Following 24 hours treatment with eugenol (0.5, 0.9, 1.2 mM) MCF-7 cells (500,000 cells in petri dish) were digested with 1x trypsin and collected by centrifugation (1500 rpm) for 5 min at 4 °C. Cells were then washed with ice cold PBS, fixed in ethanol and stained for 10 min with PBS containing propidium iodide (30 μl, 1 mg/ml) and RNase A (100 μl, 200 μg/ml). Cell cycle analysis was performed using GuavaEasy Cyte8 Flow cytometer. Each sample was analyzed for subG0 population.

### Apoptosis quantification using annexin V/PI staining

Apoptosis was also assessed following the instruction manual of Annexin V-FITC Apoptosis Detection Kit (abcam, USA). Annexin V is a protein that binds phosphatidylserine residues exposed on the surface of apoptotic cells. MCF-7 cells (100,000 cells in 6 wells plate) were treated with 0.9 mM eugenol for 24 hours. Cells were then trypsinized, rinsed twice with PBS, re-suspended in 200 μl of 1x binding buffer and labeled (5 min) with FITC-conjugated Annexin V (2 μl) antibody and propidium iodide (2 μl). Samples were immediately analyzed with GuavaEasy Cyte8 Flow cytometer. The Annexin V-FITC^−^/PI^−^ cell population was considered normal, whereas the Annexin V-FITC^+^/PI^−^ and Annexin V-FITC^+^/PI^+^ were indicative of early and late apoptotic cells.

### Statistical analysis

Results are reported as the mean ± standard error of the Mean (SEM) while indicating in the Figure legends, the number of determinations from the different number of experiments. The normality test for a small size sample was performed using the Shapiro-Wilk test; a p-value greater than 0.05 indicates normal distribution of the data. Statistical significance was then analyzed using the One-way Analysis of Variance followed by a Tukey-Kramer multiple comparison test. A p-value less than 0.05 is considered significant.

## Additional Information

**How to cite this article**: Al Wafai, R. *et al*. Chemosensitivity of MCF-7 cells to eugenol: release of cytochrome-c and lactate dehydrogenase. *Sci. Rep.* **7**, 43730; doi: 10.1038/srep43730 (2017).

**Publisher's note:** Springer Nature remains neutral with regard to jurisdictional claims in published maps and institutional affiliations.

## Supplementary Material

Supplementary Dataset 1

## References

[b1] Jaganathan S. K. & Supriyanto E. Antiproliferative and molecular mechanism of eugenol-induced apoptosis in cancer cells. Molecules 17, 6290–6304, doi: 10.3390/molecules17066290 (2012). 22634840 PMC6268974

[b2] Rastogi S. C., Johansen J. D. & Menne T. Natural ingredients based cosmetics. Content of selected fragrance sensitizers. Contact Dermatitis 34, 423–426 (1996). 8879930 10.1111/j.1600-0536.1996.tb02246.x

[b3] Lairungruang K., Itharat A. & Panthong S. Antimicrobial activity of extracts from a Thai traditional remedy called Kabpi for oral and throat infection and its plant components. J.Med. Assoc. Thai. 97 Suppl 8, S108–115 (2014). 25518302

[b4] Kayaoglu G., Erten H., Alacam T. & Orstavik D. Short-term antibacterial activity of root canal sealers towards Enterococcus faecalis. Int. Endod. J. 38, 483–488, doi: 10.1111/j.1365-2591.2005.00981.x (2005). 15946270

[b5] Benencia F. & Courreges M. C. *In vitro* and *in vivo* activity of eugenol on human herpesvirus. Phytother. Res. 14, 495–500 (2000). 11054837 10.1002/1099-1573(200011)14:7<495::aid-ptr650>3.0.co;2-8

[b6] Ogata M., Hoshi M., Urano S. & Endo T. Antioxidant activity of eugenol and related monomeric and dimeric compounds. Chem. Pharm. Bull. (*Tokyo*). 48, 1467–1469 (2000). 11045452 10.1248/cpb.48.1467

[b7] Pisano M. . Antiproliferative and pro-apoptotic activity of eugenol-related biphenyls on malignant melanoma cells. Mol Cancer 6, 8, doi: 10.1186/1476-4598-6-8 (2007). 17233906 PMC1785384

[b8] Asha M. K., Prashanth D., Murali B., Padmaja R. & Amit A. Anthelmintic activity of essential oil of Ocimum sanctum and eugenol. Fitoterapia 72, 669–670 (2001). 11543966 10.1016/s0367-326x(01)00270-2

[b9] Opdyke D. L. Monographs on fragrance raw materials. Food Cosmet. Toxicol. 13, 545–554 (1975). 53171 10.1016/0015-6264(75)90011-5

[b10] Hinnen C., Ranchor A. V., Baas P. C., Sanderman R. & Hagedoorn M. Partner support and distress in women with breast cancer: The role of patients’ awareness of support and level of mastery. Psychology & health 24, 439–455, doi: 10.1080/08870440801919513 (2009). 20205004

[b11] Sarrami N., Pemberton M. N., Thornhill M. H. & Theaker E. D. Adverse reactions associated with the use of eugenol in dentistry. Br. Dent. J. 193, 257–259, doi: 10.1038/sj.bdj.4801539 (2002). 12353045

[b12] Barkin M. E., Boyd J. P. & Cohen S. Acute allergic reaction to eugenol. Oral Surg. Oral Med. Oral Pathol. 57, 441–442 (1984). 6584843 10.1016/0030-4220(84)90166-x

[b13] Vilaplana J., Grimalt F., Romaguera C. & Conellana F. Contact dermatitis from eugenol in mouthwash. Contact Dermatitis 24, 223–224 (1991). 10.1111/j.1600-0536.1991.tb01703.x 1868706

[b14] Murakami Y. . Comparative inhibitory effects of magnolol, honokiol, eugenol and bis-eugenol on cyclooxygenase-2 expression and nuclear factor-kappa B activation in RAW264.7 macrophage-like cells stimulated with fimbriae of Porphyromonas gingivalis. In Vivo 26, 941–950 (2012). 23160676

[b15] da Silva P. T. . Cytotoxicity evaluation of four endodontic sealers. Braz. Dent. J. 19, 228–231 (2008). 18949296 10.1590/s0103-64402008000300010

[b16] Huang F. M., Tai K. W., Chou M. Y. & Chang Y. C. Cytotoxicity of resin-, zinc oxide-eugenol-, and calcium hydroxide-based root canal sealers on human periodontal ligament cells and permanent V79 cells. Int. Endod. J. 35, 153–158 (2002). 11843970 10.1046/j.1365-2591.2002.00459.x

[b17] Ho Y. C., Huang F. M. & Chang Y. C. Mechanisms of cytotoxicity of eugenol in human osteoblastic cells *in vitro*. Int. Endod. J. 39, 389–393, doi: 10.1111/j.1365-2591.2006.01091.x (2006). 16640638

[b18] Silva E. J. . Evaluation of cytotoxicity and up-regulation of gelatinases in human fibroblast cells by four root canal sealers. Int. Endod. J. 45, 49–56, doi: 10.1111/j.1365-2591.2011.01946.x (2012). 21910744

[b19] Pal D. . Eugenol restricts DMBA croton oil induced skin carcinogenesis in mice: downregulation of c-Myc and H-ras, and activation of p53 dependent apoptotic pathway. J. Dermatol. Sci. 59, 31–39, doi: 10.1016/j.jdermsci.2010.04.013 (2010). 20537511

[b20] Manikandan P., Vinothini G., Vidya Priyadarsini R., Prathiba D. & Nagini S. Eugenol inhibits cell proliferation via NF-kappaB suppression in a rat model of gastric carcinogenesis induced by MNNG. Invest. New Drugs 29, 110–117, doi: 10.1007/s10637-009-9345-2 (2011). 19851710

[b21] Manikandan P., Murugan R. S., Priyadarsini R. V., Vinothini G. & Nagini S. Eugenol induces apoptosis and inhibits invasion and angiogenesis in a rat model of gastric carcinogenesis induced by MNNG. Life Sci. 86, 936–941, doi: 10.1016/j.lfs.2010.04.010 (2010). 20434464

[b22] Kim G. C. . Caspases-dependent apoptosis in human melanoma cell by eugenol. Korean J. Anat. 39, 245–253 (2006).

[b23] Park B. S. . Phospho-ser 15-p53 translocates into mitochondria and interacts with Bcl-2 and Bcl-xL in eugenol-induced apoptosis. Apoptosis 10, 193–200, doi: 10.1007/s10495-005-6074-7 (2005). 15711935

[b24] Yoo C. B. . Eugenol isolated from the essential oil of Eugenia caryophyllata induces a reactive oxygen species-mediated apoptosis in HL-60 human promyelocytic leukemia cells. Cancer Lett. 225, 41–52, doi: 10.1016/j.canlet.2004.11.018 (2005). 15922856

[b25] Kim S. S. . Eugenol suppresses cyclooxygenase-2 expression in lipopolysaccharide-stimulated mouse macrophage RAW264.7 cells. Life Sci. 73, 337–348 (2003). 12757841 10.1016/s0024-3205(03)00288-1

[b26] Jaganathan S. K., Mazumdar A., Mondhe D. & Mandal M. Apoptotic effect of eugenol in human colon cancer cell lines. Cell Biol. Int. 35, 607–615, doi: 10.1042/CBI20100118 (2011). 21044050

[b27] Vidhya N. & Devaraj S. N. Induction of apoptosis by eugenol in human breast cancer cells. Indian J. Exp. Biol. 49, 871–878 (2011). 22126019

[b28] Usta J., Kreydiyyeh S., Bajakian K. & Nakkash-Chmaisse H. *In vitro* effect of eugenol and cinnamaldehyde on membrane potential and respiratory chain complexes in isolated rat liver mitochondria. Food Chem. Toxicol. 40, 935–940 (2002). 12065215 10.1016/s0278-6915(02)00071-6

[b29] Leal P. F. . Functional properties of spice extracts obtained via supercritical fluid extraction. J. Agric. Food Chem. 51, 2520–2525, doi: 10.1021/jf0260693 (2003). 12696930

[b30] Aisha A. F. . Evaluation of antiangiogenic, cytotoxic and antioxidant effects of Syzygium aromaticum L. extracts. As J of Bio Sci 4, 282–290 (2011).

[b31] Babich H., Stern A. & Borenfreund E. Eugenol cytotoxicity evaluated with continuous cell lines. Toxicol In Vitro 7, 105–109 (1993). 20732177 10.1016/0887-2333(93)90119-p

[b32] Al-Sharif I., Remmal A. & Aboussekhra A. Eugenol triggers apoptosis in breast cancer cells through E2F1/survivin down-regulation. BMC cancer 13, 600, doi: 10.1186/1471-2407-13-600 (2013). 24330704 PMC3931838

[b33] Pavithra B. Eugenol-A Review. J. Pharm. Sci and Res. 6, 153–154 (2014).

[b34] Han E. H. . Eugenol inhibit 7,12-dimethylbenz[a]anthracene-induced genotoxicity in MCF-7 cells: Bifunctional effects on CYP1 and NAD(P)H:quinone oxidoreductase. FEBS Lett. 581, 749–756, doi: 10.1016/j.febslet.2007.01.044 (2007). 17275817

[b35] Chaieb K. . The chemical composition and biological activity of clove essential oil, Eugenia caryophyllata (Syzigium aromaticum L. Myrtaceae): a short review. Phytother. Res. 21, 501–506, doi: 10.1002/ptr.2124 (2007). 17380552

[b36] Dorai T. & Aggarwal B. B. Role of chemopreventive agents in cancer therapy. Cancer Lett. 215, 129–140, doi: 10.1016/j.canlet.2004.07.013 (2004). 15488631

[b37] Hume W. R. Effect of eugenol on respiration and division in human pulp, mouse fibroblasts, and liver cells *in vitro*. J. Dent. Res. 63, 1262–1265 (1984). 6438202 10.1177/00220345840630110101

[b38] Kasugai S., Hasegawa N. & Ogura H. A simple in vito cytotoxicity test using the MTT (3-(4,5)-dimethylthiazol-2-yl)-2,5-diphenyl tetrazolium bromide) colorimetric assay: analysis of eugenol toxicity on dental pulp cells (RPC-C2A). Jpn. J. Pharmacol. 52, 95–100 (1990). 2308240 10.1254/jjp.52.95

[b39] Nohl H., Kozlov A. V., Gille L. & Staniek K. Cell respiration and formation of reactive oxygen species: facts and artefacts. Biochem. Soc. Trans. 31, 1308–1311, doi: 10.1042/ (2003) . 10.1042/bst0311308 14641050

[b40] Simon H. U., Haj-Yehia A. & Levi-Schaffer F. Role of reactive oxygen species (ROS) in apoptosis induction. Apoptosis 5, 415–418 (2000). 11256882 10.1023/a:1009616228304

[b41] Atsumi T., Iwakura I., Fujisawa S. & Ueha T. Reactive oxygen species generation and photo-cytotoxicity of eugenol in solutions of various pH. Biomaterials 22, 1459–1466 (2001). 11374444 10.1016/s0142-9612(00)00267-2

[b42] Suzuki Y., Sugiyama K. & Furuta H. Eugenol-mediated superoxide generation and cytotoxicity in guinea pig neutrophils. Jpn. J. Pharmacol. 39, 381–386 (1985). 3005730 10.1254/jjp.39.381

[b43] Li Y. & Trush M. A. Reactive oxygen-dependent DNA damage resulting from the oxidation of phenolic compounds by a copper-redox cycle mechanism. Cancer Res. 54, 1895s–1898s (1994). 8137307

[b44] Sakano K., Inagaki Y., Oikawa S., Hiraku Y. & Kawanishi S. Copper-mediated oxidative DNA damage induced by eugenol: possible involvement of O-demethylation. Mutat. Res. 565, 35–44, doi: 10.1016/j.mrgentox.2004.08.009 (2004). 15576237

[b45] McClain D. E., Kalinich J. F. & Ramakrishnan N. Trolox inhibits apoptosis in irradiated MOLT-4 lymphocytes. FASEB J. 9, 1345–1354 (1995). 7557025 10.1096/fasebj.9.13.7557025

[b46] Nitescu N. . N-acetylcysteine attenuates kidney injury in rats subjected to renal ischaemia-reperfusion. Nephrol. Dial. Transplant. 21, 1240–1247, doi: 10.1093/ndt/gfk032 (2006). 16390850

[b47] Thompson D., Constantin-Teodosiu D., Norbeck K., Svensson B. & Moldeus P. Metabolic activation of eugenol by myeloperoxidase and polymorphonuclear leukocytes. Chem. Res. Toxicol. 2, 186–192 (1989). 2562421 10.1021/tx00009a011

[b48] Thompson D. . Peroxidase-catalyzed oxidation of eugenol: formation of a cytotoxic metabolite(s). J. Biol. Chem. 264, 1016–1021 (1989). 2536013

[b49] Thompson D. C., Constantin-Teodosiu D. & Moldeus P. Metabolism and cytotoxicity of eugenol in isolated rat hepatocytes. Chem. Biol. Interact. 77, 137–147 (1991). 1991333 10.1016/0009-2797(91)90069-j

[b50] Thompson D. C., Barhoumi R. & Burghardt R. C. Comparative toxicity of eugenol and its quinone methide metabolite in cultured liver cells using kinetic fluorescence bioassays. Toxicol. Appl. Pharmacol. 149, 55–63, doi: 10.1006/taap.1997.8348 (1998). 9512727

[b51] Jeng J. H., Hahn L. J., Lu F. J., Wang Y. J. & Kuo M. Y. Eugenol triggers different pathobiological effects on human oral mucosal fibroblasts. J. Dent. Res. 73, 1050–1055 (1994). 8006231 10.1177/00220345940730050601

[b52] Meister A. Glutathione, ascorbate, and cellular protection. Cancer Res. 54, 1969s–1975s (1994). 8137322

[b53] Dekhuijzen P. N. Antioxidant properties of N-acetylcysteine: their relevance in relation to chronic obstructive pulmonary disease. Eur. Respir. J. 23, 629–636 (2004). 15083766 10.1183/09031936.04.00016804

[b54] Castro L. & Freeman B. A. Reactive oxygen species in human health and disease. Nutrition 17(161), 163–165 (2001). 10.1016/s0899-9007(00)00570-0 11240347

[b55] Richter C., Schweizer M., Cossarizza A. & Franceschi C. Control of apoptosis by the cellular ATP level. FEBS Lett. 378, 107–110 (1996). 8549813 10.1016/0014-5793(95)01431-4

[b56] Sofian M. . Screening of family members of patients with acute brucellosis in an endemic area of Iran. Iranian journal of microbiology 5, 215–219 (2013). 24475326 PMC3895557

[b57] Devi K. P., Sakthivel R., Nisha S. A., Suganthy N. & Pandian S. K. Eugenol alters the integrity of cell membrane and acts against the nosocomial pathogen Proteus mirabilis. Arch. Pharm. Res. 36, 282–292, doi: 10.1007/s12272-013-0028-3 (2013). 23444040

[b58] Oyedemi S. O., Okoh A. I., Mabinya L. V., Pirochenva G. & Afolayan A. J. The proposed mechanism of bactericidal action of eugenol, α-terpineol and g-terpinene against Listeria monocytogenes, Streptococcus pyogenes, Proteus vulgaris and Escherichia coli. Afr J Biotechnol 8, 7 (2009).

[b59] Ali S. M. . Antimicrobial activities of Eugenol and Cinnamaldehyde against the human gastric pathogen Helicobacter pylori. Annals of clinical microbiology and antimicrobials 4, 20, doi: 10.1186/1476-0711-4-20 (2005). 16371157 PMC1373661

[b60] Burkey J. L., Sauer J. M., McQueen C. A. & Sipes I. G. Cytotoxicity and genotoxicity of methyleugenol and related congeners– a mechanism of activation for methyleugenol. Mutat. Res. 453, 25–33 (2000). 11006409 10.1016/s0027-5107(00)00070-1

[b61] Rompelberg C. J., Evertz S. J., Bruijntjes-Rozier G. C., van den Heuvel P. D. & Verhagen H. Effect of eugenol on the genotoxicity of established mutagens in the liver. Food Chem. Toxicol. 34, 33–42 (1996). 8603795 10.1016/0278-6915(95)00091-7

[b62] Teng Z., Kuang X., Wang J. & Zhang X. Real-time cell analysis–a new method for dynamic, quantitative measurement of infectious viruses and antiserum neutralizing activity. J. Virol. Methods 193, 364–370, doi: 10.1016/j.jviromet.2013.06.034 (2013). 23835032

