# Supplementary material for: Chemosensitivity of MCF-7 cells to eugenol: release of cytochrome-c and lactate dehydrogenase
Source: Sci Rep. 2017 Mar 8;7:43730. doi: 10.1038/srep43730 (PMC5341120; doi:10.1038/srep43730)
Supplement: Supplementary Dataset 1 [file srep43730-s1.doc]

**Chemosensitivity of MCF-7 cells to eugenol: release of cytochrome-c and lactate dehydrogenase**

**Rana Al Wafaia, Warde El-Rabiha, Meghri Katerjia, Remi Safib, Marwan El Sabbanb, Omar El-Rifaia, Julnar Ustaa***

a Department of Biochemistry and Molecular Genetics, Faculty of Medicine, American University of Beirut, Beirut,- Lebanon.

b Department of Anatomy, Cell Biology and Physiological Sciences, Faculty of Medicine, American University of Beirut, Beirut,- Lebanon.

***Corresponding author Julnar Usta, PhD Department of Biochemistry and Molecular Genetics Faculty of Medicine American University of Beirut Beirut-Lebanon Email Address:** [**justa@aub.edu.lb**](mailto:justa@aub.edu.lb)
